# Therapeutic Potential of Curcumin as an Antimycobacterial Agent

**DOI:** 10.3390/biom11091278

**Published:** 2021-08-26

**Authors:** Nilakshi Barua, Alak Kumar Buragohain

**Affiliations:** 1 Department of Molecular Biology and Biotechnology, Tezpur University, Tezpur 784028, India; 2 Department of Microbiology, Faculty of Medicine, The Chinese University of Hong Kong, Prince of Wales Hospital, Shatin 999077, Hong Kong; 3 Department of Biotechnology, Royal Global University, Guwahati 781035, India

**Keywords:** tuberculosis, curcumin, *Mycobacterium tuberculosis*, antimycobacterial activity

## Abstract

Curcumin is the principal curcuminoid obtained from the plant *Curcuma longa* and has been extensively studied for its biological and chemical properties. Curcumin displays a vast range of pharmacological properties, including antimicrobial, anti-inflammatory, antioxidant, and antitumor activity. Specifically, curcumin has been linked to the improvement of the outcome of tuberculosis. There are many reviews on the pharmacological effects of curcumin; however, reviews of the antitubercular activity are comparatively scarcer. In this review, we attempt to discuss the different aspects of the research on the antitubercular activity of curcumin. These include antimycobacterial activity, modulation of the host immune response, and enhancement of BCG vaccine efficacy. Recent advances in the antimycobacterial activity of curcumin synthetic derivatives, the role of computer aided drug design in identifying curcumin targets, the hepatoprotective role of curcumin, and the dosage and toxicology of curcumin will be discussed. While growing evidence supports the use of curcumin and its derivatives for tuberculosis therapy, further preclinical and clinical investigations are of pivotal importance before recommending the use of curcumin formulations in public health.

## 1. Introduction

Tuberculosis (TB), a communicable disease caused by the bacillus *Mycobacterium tuberculosis* (MTB), is one of the top ten causes of death worldwide. Currently, about a quarter of the world’s population is infected with MTB [1]. TB is a major international health problem and is a leading cause of death from a single infectious agent (ranking above HIV/AIDS).

The emergence of multidrug-resistant strains of Mycobacterium tuberculosis (MTB) and the adverse effects of antituberculosis drugs have renewed the interest in natural products for the discovery of new antitubercular leads [2]. The use of new techniques for evaluation of the antimycobacterial activity has led to the identification of many natural products that demonstrate potent inhibition of MTB and, in some cases, the mechanism of action has also been determined [3,4]. Natural products that have demonstrated inhibitory effects on the growth of MTB include secondary metabolites derived from plants, marine organisms, algae bacteria, and fungi and are categorized as terpenes, steroids, alkaloids, aromatics, polyketides, peptides, [5] and their synthetic derivatives [6]. One such natural product is curcumin, a plant-derived lipophilic polyphenol that has been demonstrated to possess therapeutic benefits in multiple diseases, including arthritis [7], cancers [8], inflammation [9], liver disease [10], metabolic syndrome [11], neurodegenerative diseases [12], and obesity [13]. Recent studies present curcumin and its derivatives as promising antitubercular drugs that could be used alone or in combination with other drugs. In this review, we discuss how curcumin and its derivatives affect TB.

Curcumin is also known as turmeric yellow or diferuloylmethane, a beta-diketone [14] that constitutes 2–5% of turmeric powder [15]. Turmeric is acquired from the rhizome of a tuberous herbaceous perennial plant, *Curcuma longa* L., a member of the Zingiberaceae family. The turmeric plant is native to the Indian subcontinent, and its use can be found abundantly in the traditional medical literature of India, Pakistan, Bangladesh, Bhutan, and China [16,17].

**Figure 1:**
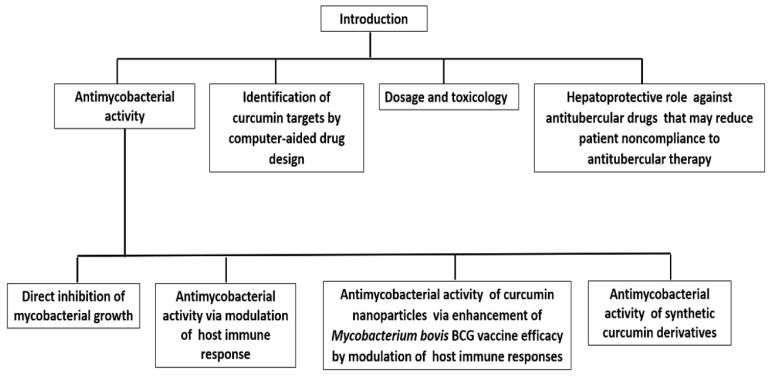
Scheme describing the overview of the review.

Pharmacological studies of curcumin suggest that it has a potent protective capacity against TB [15]. Therefore, the explanation of the hypothesis that curcumin and its derivatives have an antitubercular role is no longer in doubt. In this manuscript, we attempt to update the knowledge and review a short historical overview of the past eight year’s findings on the antitubercular activity of curcumin and its derivatives. The review summarizes and discusses the studies that provide evidence of the potential development of curcumin-based antitubercular drugs. Figure 1 depicts an overview of our review.

## 2. Physical, Chemical, and Biochemical Properties of Curcumin

**Figure 2:**
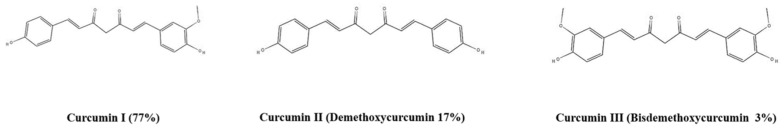
Phytoconstituents of *Curcuma longa*. Curcumin I (77%), curcumin II (demethoxycurcumin 17%), and curcumin III (bisdemethoxycurcumin 3%) constitute the curcuminoids isolated from turmeric.

Curcumin I (77%), curcumin II (demethoxycurcumin 17%), and curcumin III (bisdemethoxycurcumin 3%) constitute the curcuminoids isolated from turmeric (Figure 2). The natural analogs demethoxycurcumin (DMC) and bisdemethoxycurcumin (BDMC) possess biological activity similar to that of curcumin [18]. In addition to curcuminoids, turmeric comprises carbohydrate (69.4%), protein (6.3%), fat (5.1%), minerals (3.5%), and moisture (13.1%) [19]. Curcumin is soluble in solvents like acetone, methanol, and ethanol. Curcumin is a keto-enol tautomeric compound that exists in a predominant keto-form in acid or neutral solutions and the enol-form in alkali solutions exhibiting the properties of metal ion chelator [20]. Curcumin has substantial protective and preventive effects against various diseases such as cancer, autoimmune, neurological, metabolic, lung, liver, and cardiovascular diseases as the keto-enol forms endow curcumin with antiangiogenic, anti-inflammatory, antimicrobial, antimutagenic, antioxidant, and antiplatelet aggregation properties. Due to its light sensitivity, it is recommended that curcumin-containing samples be protected from light [21,22,23].

### 2.1. Curcumin as an Antimycobacterial Agent

Curcumin presents itself as a novel and robust strategy to combat the virulence and antibiotic resistance of *Mycobacterium abscessus*. Curcumin at minimum inhibitory concentrations (MIC) of 128 μg/mL exhibited synergistic activity with amikacin, ciprofloxacin, clarithromycin, and linezolid against a clinical strain of *M. abscessus*. Curcumin significantly (*p* < 0.05) reduced the motility of *M. abscessus* at 1/8th the concentration of MIC. The biofilm formed by MTB contributes to its virulence and drug tolerance, and at a concentration four times higher than the MIC curcumin completely inhibited day four and eight mature biofilms in terms of biomass reduction [24].

### 2.2. Curcumin Exhibits Antitubercular Activity via Modulation of the Host Immune Response

**Figure 3:**
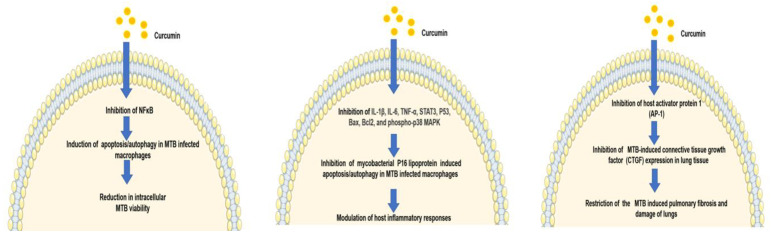
Various mechanisms of curcumin antitubercular activity via modulation of the host immune response. The figure was produced using Servier Medical Art (http://smart.servier.com/ accessed on 1 February 2021).

Curcumin modulates the clearance of MTB by macrophages via the induction of apoptosis. Preincubation of human THP-1 monocytes and primary human alveolar macrophages with curcumin at a concentration of 10, 30, and 50 μM for 1 h before infection with MTB H37Rv have reduced the burden of intracellular MTB. Compared to the control cells, which were incubated with 0.05% DMSO, incubation with curcumin exhibited a significant reduction (*p* < 0.05) in the number of MTB recovered at two and four days after infection. However, curcumin at concentrations up to 50 μM did not significantly affect MTB growth in the absence of macrophages, indicating that curcumin aids in the clearance of the MTB by enhancing macrophage activity. TUNEL assay of the MTB-infected THP-1 cells revealed that curcumin induced apoptosis in macrophages in a dose-dependent manner. The enhanced clearance of MTB in the differentiated THP-1 human monocytes and primary human alveolar macrophages was by curcumin-induced caspase-3-dependent apoptosis and autophagy [25]. MTB activates NFκB, causing inhibition of both apoptosis and autophagy in infected human macrophages, and evades host clearance [26,27,28]. Curcumin induces an anti-MTB cellular response in the macrophages via inhibition of the p65 NFκB, binding to its consensus oligonucleotide and activation of the NFκB and induction of apoptosis, causing a substantial reduction in bacterial viability [25]. Figure 3 depicts the various mechanisms of curcumin antitubercular activity via modulation of the host immune response.

However, the results of another study contradict this, showing that curcumin at low doses (10 and 20 μM) protected MTB infected macrophages from 19-kDa lipoprotein (P19) and induced inflammation and apoptosis. P19, a virulence factor secreted by the MTB, activates Toll-like receptor 2 (TLR2) and mitogen-activated protein kinases (MAPKs) in MTB infected macrophages. However, curcumin affects the extracellular signal-regulated protein kinase (ERK), showing that curcumin inhibits apoptosis of macrophages mediated by P19 by regulating the JNK pathway [29]. Blocking of the p38 mitogen-activated protein kinases (p38 MAPK) signaling pathway in human macrophages cell line WBC264-9C with the concomitant treatment of curcumin at a concentration of 20 and 40 μmol/L and the MTB P19 revealed that curcumin modulates the inflammatory responses and apoptosis induced by P19. Curcumin attenuated the P19-induced growth inhibition significantly (*p* < 0.01), as shown by significantly lowered expression of the cytokines IL-1β, IL-6, TNF-α, the signal transducer and transcription activator 3 (STAT3), and apoptotic proteins P53, Bax, Bcl2, and phospho-p38 MAPK expression measured in the presence and absence of the antagonist of p38 MAPK [30]. Overall, antitubercular activity of curcumin via modulation of the host immune response remains unclear and needs to be further studied to elucidate its protective role in MTB infected macrophages.

Acetamiprid (ACE) is an insecticide belonging to the neonicotinoid family, which induces immunosuppression of specific humoral and cellular responses against the MTB virulence factor CFP32. Curcumin treatment at the dose of 100 mg/kg partially restored this Acetamiprid (ACE) mediated immunosuppression in Swiss Albino mice after exposure to ACE at the dose of 5 mg/kg. Treatment for 61 days showed significant (*p* < 0.05) restoration in the humoral immune response when assessed by ELISA with anti-rCFP32 antibody concentrations in the serum compared to Swiss Albino mice treated with ACE only. The cellular immune response was also partially restored when assessed by the cellular proliferation of the splenocytes stimulated by rCFP32, showing that the use of curcumin could be a new potential strategy to reduce the ACE-induced immunotoxicity, especially in the population involved in the agricultural sector [31].

Upon infection by MTB in the lung alveoli, the host immune system responds via the formation of granulomas by macrophages, epithelial cells, and fibroblasts [32]. The fibroblast plays a major role in tissue remodeling during the formation of the granuloma. Cavitation of the lung tissue caused by TB leads to the formation of fibrotic tissues by myofibroblasts and epithelial cells around the cavity [33], thus inducing pulmonary fibrosis and a reduction in the function of the respiratory system [34]. The connective tissue growth factor (CTGF) is a secreted cysteine-rich protein that plays a crucial role in lung fibrosis. MTB induces CTGF expression through TLR2 but not TLR4 through the promoter region at 2747 to 2184 bp containing activator protein 1 (AP-1) and the signal transducer activator of transcription binding sites. Curcumin, being an AP-1 inhibitor, has been reported to play a significant role in restricting MTB induced pulmonary fibrosis by restraining the MTB-induced CTGF expression [35].

### 2.3. Curcumin Nanoparticles Enhance Mycobacterium bovis BCG Vaccine Efficacy by Modulating Host Immune Responses

The only available vaccine against TB that is effective against disseminated and meningeal TB in young children is *Mycobacterium bovis* bacillus Calmette-Guérin (BCG) [36]. The main drawback of BCG is its inefficacy in protecting against adult pulmonary TB, which is mainly due to the decline with time in the host-protective immune responses that it induces [37]. Curcumin nanoparticles (nanocurcumin) enhance the functions of dendritic cells, macrophages, Langerhans cells, and B cells, which are the host antigen-presenting cells (APC) and modulate the host immune responses via autophagy, costimulatory activity, and the production of inflammatory cytokines. Further, nanocurcumin enhanced the efficacy of BCG in the induction of T cells with long-lasting central memory T (TCM) cells of the Th1 and Th17 lineages, which elevate host immune protection against MTB infection. Nanocurcumin reduced the bacterial burden (*p* ≤ 0.05) of MTB H37Rv-infected peritoneal macrophages at a concentration of 60 nM in a time-dependent fashion. Nanocurcumin treatment at 60 nM significantly (*p* ≤ 0.05) enhanced cellular activation, autophagy of the macrophages, and production of TNF-α (the cytokine involved in the production of NO and other free radicals), which together clear MTB. The cytokine IL-10 suppresses protective immune responses by the downregulation of the expression of major histocompatibility complex (MHC) class II (MHCII) and costimulatory molecules. Nanocurcumin significantly (*p* ≤ 0.05) downregulated the levels of IL-10 in H37Rv-infected macrophages. The efficacy of the BCG vaccine was also enhanced by nanocurcumin in the C57BL/6 murine TB infection model. Subcutaneous administration of the BGC vaccine and subsequent treatment with nanocurcumin for 30 days, followed by a resting period of 30 days, causes a significant (*p* ≤ 0.05) reduction in bacterial burden and reduction of granulomatous regions in the organs of the mice when challenged with MTB-H37Rv aerosol at a low dose of approximately 110 Colony Forming Units (CFU) showing the enhancement of the BCG vaccine [38].

Curcumin nanoparticles also exhibited fivefold enhanced bioavailability. By themselves, curcumin nanoparticles inhibited the growth of the MTB H37Rv strain by at best 1-log in the mice and accelerated the clearance of the MTB from the lung and spleen of BALB/c mice by promoting an antitubercular response, which in turn reduced the duration of therapy. Curcumin nanoparticles restored the isoniazid (INH)-induced suppression in antigen-specific cytokine, the proliferation of T cells suppressed by INH, and reduced hepatotoxicity in mice induced by antitubercular antibiotics. Far more intriguing, curcumin nanoparticle treatment raised the total number of splenocytes, enhanced the frequency and activation of both CD4^+^ and CD8^+^ T cells, and also reduced the risk for TB reactivation and reinfection [39].

To improve intramacrophage delivery and improved MTB clearance, rifampicin (RIF) and curcumin were co-encapsulated in polymeric nanoparticles with an average size of ~400 nm, low polydispersity, and zeta potential of 26.89 ± 2.9 mV. Both rifampicin and the curcumin were released in the lysosomal fluid, indicating that the drugs were released by the nanoparticles only after the macrophage internalization. These nanoparticles were nontoxic to RAW 264.7 macrophages and increased the drug internalization 1.5-fold compared to free drugs as determined by confocal microscopy. Encapsulation did not affect the drug properties as confirmed by the comparable minimum inhibitory concentration of free RIF, free curcumin, and nanoparticle encapsulated RIF and curcumin. The nanoencapsulation method is suggested to be a promising tool to tackle TB as high efficacy was exhibited by the RIF-CUR nanoparticles, which cleared the MTB infected macrophages at 25× MIC (98.03 ± 2.5%) as well as complete clearance above 50× MIC [40].

## 3. Antimycobacterial Activity of Curcumin Synthetic Derivatives

Although curcumin is a promising antimycobacterial agent, its use in clinical and pharmaceutical studies has been decelerated by its poor chemical stability [41,42]. In addition, curcumin has low oral bioavailability and is rapidly excreted due to its poor absorption and extensive intestinal and first-pass metabolism [43]. Therefore, efforts have been made to synthesize [44] and identify more stable analogues and potent antimycobacterial analogues. as listed in Table 1.

**Figure 4:**
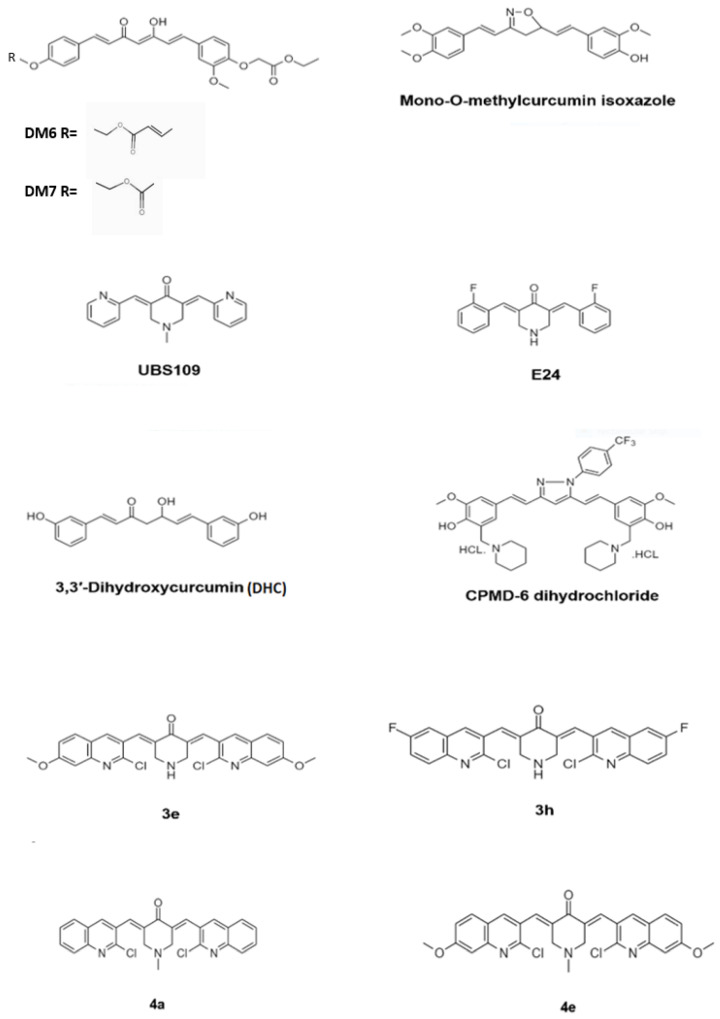
Chemical structure of the curcumin derivatives that have exhibited potent antimycobacterial activity.

Demethoxycurcumin, one of the curcuminoids, exhibited potent antimycobacterial activity against MTB H37Rv strain at 200 μg/mL. Synthetic derivatives yielded potent antimycobacterial agents, which exhibited a considerable activity with the MIC value of 7.8 μg/mL. Four derivates of demethoxycurcumin, DM4–DM7, were synthesized by chemical modification at its phenolic hydroxy positions. The derivative DM6 exhibited potent antitubercular with a MIC of 7.812 μg/mL. Derivative DM7 showed moderate activity with a MIC of 125 μg/mL, whereas the derivatives DM1, DM3, DM4, and DM5 were inactive against MTB even at the concentration of 250 μg/mL. The derivatives DM6 and DM7 possessed increased lipophilicity due to the presence of fatty acid ester chains at the phenolic hydroxy groups. The increased lipophilicity may have endowed the two derivatives DM6 and DM7 with better antitubercular activity due to the increased interaction with the lipophilic MTB cell wall causing impairment of transport of polar compounds through the outer lipid layer of mycobacteria. The lead optimization of these two derivatives may lead to the identification of antitubercular drug candidates in the future [46]. Figure 4 depicts the chemical structure of the derivatives of curcumin.

Out of 55 isoxazole synthetic analogues of the curcuminoids, mono-O-methylcurcumin isoxazole exhibited the potent antimycobacterial activity with the MIC 0.09 μg/mL, which is 1131-fold more active than the parent compound curcumin and approximately 18 and 2-fold more active than the antimycobacterial drugs kanamycin and isoniazid, respectively, against MTB H37Ra and the clinical isolates of MDR-TB obtained from Ramathibodi Hospital, Mahidol University, Bangkok, Thailand. Mono-*O*-methylcurcumin isoxazole also exhibited high activity against the multidrug-resistant MTB clinical isolates, with MICs of 0.195–3.125 μg/mL. The isoxazole ring and two unsaturated bonds on the heptyl chain of the curcuminoid analogue are responsible for the antimycobacterial activity. The biological activity was enhanced by the *para*-alkoxyl group on the aromatic ring, attached in close proximity to the nitrogen function of the isoxazole ring in addition to the free *para*-hydroxyl group on another aromatic ring [47].

A series of eight mono-carbonyl analogues of curcumin were synthesized to increase the bioavailability of curcumin and tested the antimycobacterial activity against MTB and *M. marinum* (MM). In the initial screening using the disk diffusion assay, out of the eight analogues, seven exhibited an antimycobacterial activity at a concentration of 100 mM. The analogue UBS-109 exhibited the highest activity against MM, with an inhibition zone of 5.7 ± 0.3 mm. The analogue U2-260 exhibited the lowest activity with 1.4 ± 0 mm. The analogue ECMN-951 did not exhibit antimycobacterial activity. Using liquid culture, the IC_50_ was reported to be 10 mM for UBS-109 and 25 mM for the analogue E-24. The analogue UBS-109 exhibited potent antimycobacterial activity against MTB H37Rv and Beijing F2 with an IC_50_ of ~10 μM and 20 μM, respectively. However, the inhibitory effect of the E-24 was much lower in comparison to UBS-109. However, these curcumin analogues did not exhibit synergistic effects between the monocarbonyl analogues and RIF on inhibiting mycobacterial growth. The structure–activity analysis showed that the Michael acceptor properties of the analogues are critical for antimycobacterial activity [48].

A synthetic molecule CPMD-6-dihydrochloride, exhibiting potent antimycobacterial activity, was identified from a series of 21 curcumin–pyrazole–mannich derivatives. The bacteriostatic, bactericidal synergy with first-line antituberculosis drugs against MTB H37Rv, drug-resistant MTB strains, *M. forutitum* and *M. abscessus* was investigated. The in vivo efficacy of the derivatives was evaluated in a BALB/c mice model of MTB infection. The compound CPMD-6-dihydrochloride exhibited promising antimycobacterial activity with a MIC 2 μg/mL against MTB H37Rv, drug-sensitive as well as drug-resistant mycobacterial strains compared to curcumin which exhibits a MIC of 16 μg/mL against MTB H37Rv. While curcumin did not exhibit activity against *M. forutitum* and *M. abscessus* (MIC *>* 64 μg/mL), CPMD-6 dihydrochloride exhibited potent activity with a MIC of 16 μg/mL. Interestingly, CPMD-6 dihydrochloride antimicrobial activity was specific to *Mycobacterium* sp. and did not show any antimicrobial activity against the *Enterococcus faecium*, *Staphylococcus aureus*, *Klebsiella pneumoniae*, *Acinetobacter baumannii*, *Pseudomonas aeruginosa*, and *Enterobacter* spp. (ESKAPE) panel. CPMD-6-dihydrochloride also exhibited strong synergy with the current first-line antimycobacterial drugs with a fractional inhibitory concentration (FIC) index of 0.5 for rifampin (RIF), INH, and 0.37 for ethambutol (EMB), but did not exhibit any interaction with streptomycin (STR) with a FIC index of 0.75 for STR. Three-week treatment with 25 mg/kg of CPMD-6-dihydrochloride showed a significant reduction in the bacterial load in the lung of infected six-week-old BALB/c mice by 1.06 log10 in comparison to 100 mg/kg of EMB treated group. CPMD-6-dihydrochloride also reduced bacterial counts by 0.63 log10 while EMB reduced by 0.51 log10 in the spleen, demonstrating that CPMD-6 dihydrochloride has superior efficacy with respect to a reduction in mycobacterial CFU at one-fourth of the dosage in comparison to EMB in the murine MTB infection model [45].

In a series of twelve diphenylheptanoid-derived synthetic curcuminoid analogues, 3,3′-dihydroxycurcumin (DHC) exhibited promising antimycobacterial activity against MTB with a MIC of 156 μg/mL. DHC is more stable than curcumin in phosphate buffer (pH 7.4) and acetate buffer (pH 5.0) for 24 h at 37 °C. The cell division protein FtsZ may be the target for DHC due to the fact that curcumin exhibits this mode of antibacterial action. DHC exhibited moderate toxicity in human cells from the liver (tumorigenic HepG2 cell line) and lung (tumorigenic A549 cell) and normal MCR-5 cell lines with the IC_50_ values ranging from 9.6 to 10.6 μg/mL, respectively, which is slightly more toxic then curcumin. Curcumin exhibited IC_50_ values ranging from 15.5 μg/mL to 32.3 μg/mL. No significant difference in IC_50_ in the lungs and liver cell lines indicated that the biotransformation capacity of hepatocytes did not affect the cytotoxicity of DHC. DHC toxicity in normal human fibroblasts (MCR-5) and adenocarcinoma cells (A549) showed no significant difference, indicating that tumorigenic genes and proteins did not affect its toxicity and alkaline comet assay revealed that DHC could not induce DNA damage in the A549 cell line [49].

## 4. Identification of Curcumin Targets in Mycobacterium by Computer-Aided Drug Design

The systematic study of the targets and mechanisms of natural products through traditional assay-based methods is a time consuming and costly process because of the difficulty of extraction and activity testing. Virtual in silico screening is anticipated to be an alternative approach for low-cost and rapid analysis of natural products and efficient identification for their targets. Molecular docking is a potent virtual screening tool in rational drug design that could be utilized to investigate and identify ligands and the potential targets that could be extended to analyze the structural and molecular mechanics of the binding between the ligand and protein.

The structure-based drug design (SBDD) approach, a category of computer-aided drug design, has contributed to the introduction of lead compounds into clinical trials and to numerous drug approvals [51,52]. Molecular docking studies also revealed universal stress protein (USP), a novel therapeutic target of MTB, to be the target of curcumin. The docking of curcumin to the USP protein was done using AutoDock 4.2. Curcumin was shown to form a hydrogen bond ARG 136 (1.8 Å) and two ionic bonds with a carboxyl group of curcumin with LEU 130 (3.3 Å) and ASN 144 (3.4 Å) indicating possible new curcumin analogues for future therapy to downregulate USP [53]. The bottom-up systems biology approach revealed that aspartate-β-semialdehyde dehydrogenase (ASD), dihydrodipicolinate reductase and diaminopimelate decarboxylase are potential therapeutic targets of MTB infections. In silico molecular docking study using AutoDock 4.2.6 of these targets, which was prioritized based on flux and elementary mode analysis using direct mathematical modeling of the relevant metabolic pathways, identified curcumin as ASD inhibitors [54]. Computational models revealed that the synthetic derivative of curcumin, monoacetylcurcumin, binds to the specific BRCT domain of the essential enzyme *Mtu*LigA for MTB. *Mtu*LigA is unique to MTB, thus making it a promising drug target [55]. In vitro experiments to investigate the inhibitory activity of curcumin monoacetyl derivative against BRCT domain-containing DNA polymerase λ [56] proved to be nearly twice as effective (IC_50_ 3.9) as curcumin (IC_50_ 7.0 μM). These predicted targets are summarized in Table 2.

## 5. The Dosage and Toxicology of Curcumin

The FDA has confirmed that curcumin is “generally recognized as safe” without any toxic effects. The adequate daily intake (ADI) value of curcumin is 0–3 mg/kg. Twelve grams per day intake of curcumin has no harmful effects on healthy individuals [57,58]. However, in vitro analysis of the effect of curcumin on LS180 cells for P-glycoprotein (P-gp) induction/inhibition and CYP3A4 inhibition in a single platform showed curcumin should not be administered with bedaquiline (TMC-207), a recently approved drug for the treatment of multidrug-resistant tuberculosis (MDRTB), because both bedaquiline and curcumin are substrates for both P-gp and CYP3A4 [59].

Analysis of the antimycobacterial activity of a series of quinoline incorporated monocarbonyl curcumin analogues against MTB *H37Ra* and *M. bovis* BCG in the dormant state revealed the analogues 3e, 3h, 4a, and 4e to possess potent antitubercular activity. Analogue 3e exhibited MIC_90_ > 30 μg/mL and 2.7 μg/mL against MTB *H37Ra* and *M. bovis* BCG, respectively. The analogue 3h exhibited a MIC_90_ of > 30 and 9.2 against MTB *H37Ra* and *M. bovis* BCG, respectively. The analogue 4a revealed MIC_90_ 26.5 μg/mL and 7.3 μg/mL against MTB *H37Ra* and *M. bovis* BCG, respectively. The analogue 4e exhibited MIC_90_ of 7.8 μg/mL and 9.4 μg/mL against MTB *H37Ra* and *M. bovis* BCG, respectively. The synthetic analogues were nontoxic against MCF-7, A549, and HCT-116 cell lines. In silico target identification of the analogues by molecular docking study identified *MTB* pantothenate synthetase (MTB PS) enzyme as a target of these antitubercular agents [50].

## 6. Hepatoprotective Role of Curcumin against Anti-TB Chemotherapy (ATT)

One of the significant drawbacks of TB chemotherapy is the toxicity of the drugs used, which eventually causes liver injury in TB patients and is a major cause of patients’ noncompliance to the anti-TB chemotherapy (ATT) [60]. Reduction of the hepatoxicity induced by the ATT by using curcumin has been explored recently. The anti-TB chemotherapy (ATT) involving INH, RIF, and PYR eventually causes hepatotoxicity in approximately 11.5% of Indian patients, primarily due to oxidative stress caused by the drugs and metabolites. The accumulation of an endogenous hepatotoxin, protoporphyrin IX (PPIX), has been observed due to INH/RIF co-therapy in the L-02 cell line derived from a fetal human normal liver (L-02) and liver of PXR-humanized mice. Ferrochelatase (FECH) plays a crucial role in PPIX metabolism, and INH/RFP treatment downregulates FECH both in L-02 cells and mouse livers. Curcumin treatment at a concentration of 5 μM and 10 μM could alleviate the INH/RFP induced liver injury in L-02 cell line by induction of FECH expression and decline in PPIX levels. Cotreatment with INH (75 mg/kg, ip) plus RFP (150 mg/kg, ip) with concomitant administration of curcumin (200 mg/kg, ig) revealed that curcumin possesses a protective effect by reducing the PPIX levels and induced FECH expression in male ICR mouse livers [61].

Machado et al. reported that curcumin could be an inhibitor against efflux pumps in MTB, which endows MTB with the intrinsic resistance to a vast number of the currently available arsenal of drugs [62].

## 7. Conclusions

Numerous attempts have been made in successive years to investigate the antimycobacterial activity of curcumin and its derivatives. Each subsequent year, new synthetic derivatives, new targets, and new modes of action of curcumin have been elucidated. Animal studies, as well as human trials, have shown that curcumin and its derivatives have improved the outcome of TB infection, suggesting the potential application of curcumin to be used therapeutically in the treatment of TB. Although details of the molecular mechanism of the antitubercular activity of curcumin have not yet been fully elucidated, its activity could possibly be connected to many of the molecular pathways known to engage in the pathophysiology of TB. It can be expected that curcumin modulates a vast range of physiological processes and biochemical signaling pathways during TB. In summary, curcumin and its derivatives possess antimycobacterial activity and exhibit antitubercular activity by modulating the host immune response during infection. Curcuminoids are safe for human consumption; however, evaluation of the derivatives is obligatory. The objective of our review is to evaluate the therapeutic role of curcumin and its derivatives in TB. Our article provides proof supporting the perspective that curcumin could be considered a potent antitubercular agent and could be used for derivation of novel drugs for protection as well as therapy of TB.

## Figures and Tables

**Table 1:** Minimum inhibitory concentration of curcumin, demethoxycurcumin, and their respective synthetic analogues against various mycobacterial strains (μg/mL).

Compound	Mycobacterial Strains	MIC (μg/mL)	Reference
Curcumin	MTB H37Rv	16	[45]
Demethoxycurcumin (DM)	MTB H37Rv	200	[46]
DM6	MTB H37Rv	7.8	
DM7	MTB H37Rv	125	
Mono-O-methylcurcumin- isoxazole	MTB H37Ra	0.09	[47]
	^a^ INH, RIF, STM-resistant MTB (clinical isolate M3)	0.195	
	^b^ INH, RIF, EMB, STM-resistant MTB (clinical isolate M4)	1.56	
	INH, RIF, STM-resistant MTB (clinical isolate M5)	3.125	
	INH, RIF-resistant MTB (clinical isolate M6)	0.39	
	INH, RIF, EMB, STM-resistant MTB (clinical isolate M8)	3.125	
	INH, RIF-resistant MTB (clinical isolate M11)	3.125	
	INH, RIF, EMB-resistant MTB (clinical isolate M16)	1.56	
	INH, RIF, EMB, STM-resistant MTB (clinical isolate M21)	3.125	
	INH, RIF-resistant MTB (clinical isolate M22)	0.39	
	INH, RIF-resistant MTB (clinical isolate M27)	0.195	
	^c^ INH, RIF, STM, OFX, CIP-resistant MTB (clinical isolate M46)	1.56	
	INH, RIF, STM, OFX, CIP-resistant MTB (clinical isolate M48)	1.56	
	INH, RIF, STM, OFX, CIP-resistant MTB (clinical isolate M53)	3.125	
UBS-109	*M. marinum*	10 mM *	[48]
	MTB H37Rv	~10 μM *	
EF-24	MTB Beijing F2	20 μM *	
	*M. marinum*	25 mM *	
CPMD-6- dihydrochloride	MTB H37R	2	[45]
	INH-resistant MTB ATCC 35822	2	
	RIF-resistant MTB ATCC 35838	2	
	STM-resistant MTB ATCC 35820	2	
	ETB-resistant MTB ATCC 35837	2	
	*M. fortuitum* ATCC 6841	16	
	*M.abscessus* ATCC 19977	16	
3,3′-Dihydroxycurcumin	MTB	156	[49]
Quinolidene based monocarbonyl curcumin analogue 3e	MTB	>30 ^#^	[50]
	*M. bovis* BCG	2.7 ^#^	
Quinolidene based monocarbonyl curcumin analogue 3h	MTB	>30 ^#^	
	*M. bovis* BCG	9.2 ^#^	
Quinolidene based monocarbonyl curcumin analogue 4a	MTB	26.5 ^#^	
	*M. bovis* BCG	7.3 ^#^	
Quinolidene based monocarbonyl curcumin analogue 4e	MTB	7.8 ^#^	
	*M. bovis* BCG	9.4 ^#^	

* IC_50_ of UBS-109 EF-24 against the mycobacterial strains; ^a^ INH, isoniazid; RIF, rifampicinn; STM, streptomycin; ^b^ EMB, ethambutol; ^c^ OFX, ofloxacin; CIP, ciprofloxacin; ^#^ MIC_90_.

**Table 2:** Predicted targets of Curcumin and its analogues by computer-aided drug design.

Compound	Predicted Mycobacterial Target	Reference
Curcumin	Universal stress protein (USP)	[53]
	Aspartate-β-semialdehyde dehydrogenase (ASD)	[54]
	Dihydrodipicolinate reductase	
Monoacetylcurcumin	*M. tuberculosis* NAD+-dependent DNA ligases (MtuLigA)	[55]
	BRCT domain-containing DNA polymerase λ	[56]
Quinolidene based monocarbonyl curcumin analogues 3e, 3h, 4a and 4e	Pantothenate synthetase (MTB PS)	[50]
